# Multilayer Watertight Closure to Address Adverse Events From Primary Total Knee and Hip Arthroplasty: A Systematic Review of Wound Closure Methods by Tissue Layer

**DOI:** 10.1016/j.artd.2021.05.015

**Published:** 2021-07-08

**Authors:** Mark A. Snyder, Brian P. Chen, Andrew Hogan, George W.J. Wright

**Affiliations:** a Orthopaedic Center of Excellence, Good Samaritan Hospital, Cincinnati, OH, USA; b Health Economics and Market Access, Ethicon, Inc, A Johnson & Johnson Company, Somerville, NJ, USA; c Value & Evidence Services, CRG-EVERSANA Canada Inc, Burlington, Ontario, Canada

**Keywords:** Systematic review, Total knee arthroplasty, Total hip arthroplasty, Wound closure, Surgical site infections

## Abstract

**Background:**

Wound closure is a key, and often underrecognized, component of hip and knee arthroplasty. Methods for wound closure are an important consideration to better avoid wound-related adverse events; however, there is a lack of consensus on optimal methods. The objective of the following review was twofold: to characterize the wound closure methods used by layer in the total knee arthroplasty and total hip arthroplasty literature and summarize optimal wound-healing strategies to address the risk of adverse events.

**Methods:**

A systematic literature review was performed to identify total knee arthroplasty and total hip arthroplasty randomized controlled trials and nonrandomized studies reporting wound closure methods by layer and wound-healing adverse events (including superficial, deep, or periprosthetic joint infections, wound dehiscence, or prolonged wound drainage). Studies on revision procedures were excluded. Wound closure methods and adverse events were summarized qualitatively as meta-analyses were not possible because of study heterogeneity.

**Results:**

Forty studies met the inclusion criteria: 22 randomized controlled trials and 18 observational studies. Across studies, 6 categories and 22 unique techniques for closure were identified. Conventional closure methods exhibited large ranges of adverse event rates. Studies of multilayer barbed sutures with topical skin adhesives and polyester mesh or multilayer antimicrobial sutures reported narrow ranges of adverse events rates.

**Conclusions:**

Considerable variability exists for wound closure methods, with a wide range reported in adverse events. Recent technologies and methods for standardized watertight, multilayer closure show promise for avoiding adverse events and unnecessary health-care costs; however, higher quality, comparative studies are required to enable future meta-analyses.

**Level of Evidence:**

Therapeutic Level IV. See Instructions for Authors for a complete description of levels of evidence.

## Introduction and background

Wound closure is a key, and often underrecognized, component of hip and knee arthroplasty [1,2]. Considering an estimated 1 million lower extremity total joint replacement procedures are performed annually in the United States (US), this presents an important opportunity for improving patient outcomes decayed by adverse events and early readmissions linked to suboptimal wound closure methods [3,4]. For example, a recent American College of Surgeons-National Surgical Quality Improvement Program database analysis of 169,406 patients with total joint arthroplasty found the rate of overall complications was 8% for outpatient and 16% for inpatient procedures [5].

One of the most costly and potentially avoidable adverse events after hip or knee arthroplasty is surgical site infection (SSI) [6]. The Centers for Disease Control and Prevention (CDC) National Healthcare Safety Network published criteria for the documentation of SSIs with categories including superficial and deep infections, depending on the affected tissue layers [7]. In CDC guidelines, the reported cost of SSIs range from $10,443 (2005 US dollars [USD]) to $25,546 (2002 USD) per infection [8]. Within arthroplasty procedures, another classification of infections includes periprosthetic joint infections (PJIs), defined as infections involving the joint prosthesis and adjacent tissue [9]. Costs associated with a prosthetic joint implant infection have been reported to be substantially higher, exceeding $90,000 in some cases [8]. Optimal wound closure methods can help to reduce postoperative adverse events—including SSIs and PJIs—thereby potentially lowering excess health-care resource use and costs [10].

Substantial variability in wound closure methods characterizes hip and knee arthroplasty literature, clearly demonstrating the lack of universal recommendations for optimal wound closure. Previous literature reported on adverse events associated with different wound closure techniques in hip and knee arthroplasty; however, these studies have several limitations. First, they focus on comparing one or 2 methods of wound closure, rather than the range of methods available [11,12]. Second, they do not provide consensus on the methods for use in both knee and hip arthroplasties [2,10]. Third, they focus on adverse events associated with one tissue layer, missing the full picture of multilayer closure [10]. The objective of this study was to conduct a qualitative systematic review of wound closure for hip and knee arthroplasties that characterize the various types of wound closure methods and dressings used in practice, which wound closure methods are used within different tissue layers, and reported rates of adverse events related to wound closure (ie, SSI, deep infection or PJI, prolonged wound drainage, and dehiscence).

## Material and methods

**Figure 1:**
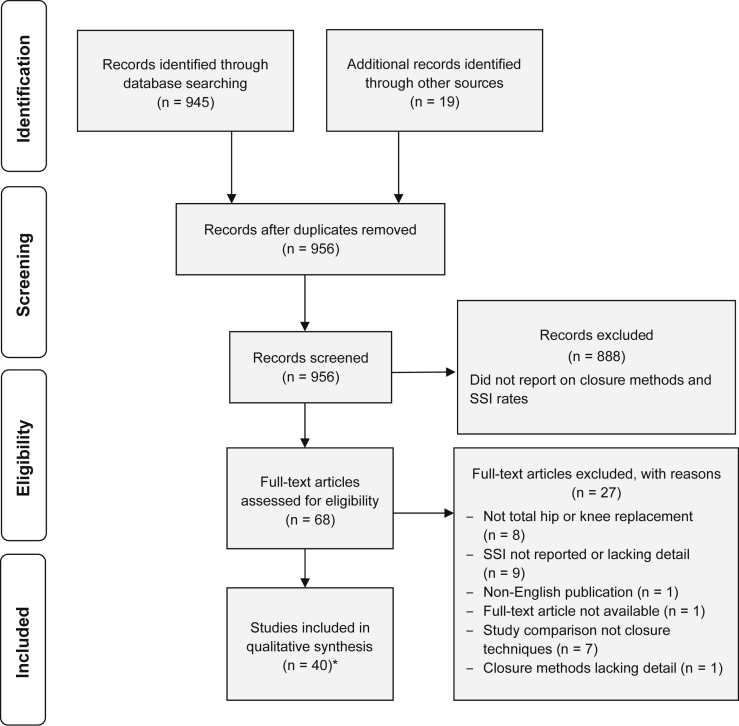
PRISMA flow diagram. ∗Note that 40 studies were included after full-text screening, but a correction was identified for Sundaram *et al.,* 2020 which was not included in the final number of included studies but was considered during data extraction.

A systematic search was performed in MEDLINE via the PubMed interface, EMBASE, and the Cochrane Library for the period of January 1, 2000, through August 28, 2020. An example search strategy for MEDLINE can be found in Appendix A. The search strategy was adapted to account for differences in database structures. To supplement electronic searches, a manual search was performed of the reference lists of all included studies as well as recent relevant reviews and meta-analyses. Separate publications reporting outcomes for the same or overlapping patient populations (linked or kin studies) were grouped together to avoid double-counting. One reviewer evaluated each title and abstract identified, and determined the eligibility based on the inclusion criteria, and documented rationale for exclusion. Abstracts that were included after title and abstract screening were assessed in full text by one reviewer, with excluded articles confirmed by a second reviewer and resolution of discrepancies resolved by study author consensus. Reasons for exclusion were documented (Fig. 1). The methods used in this analysis were aligned with the Preferred Reporting Items for Systematic Reviews and Meta-Analyses.

Study inclusion was determined according to the PICOS (ie, population, intervention/ comparator, outcome, and study design) criteria that were developed a priori. Briefly, the population of interest was patients who underwent primary hip and knee arthroplasty. The interventions and comparators included all methods of wound closure (eg, traditional sutures, antimicrobial sutures [ie, triclosan-coated sutures], barbed sutures, staples, topical skin adhesives [TSA]), used in both superficial and deep tissue layers, and dressings if reported. Outcomes included were superficial and deep SSI (primary; studies were required to report on SSI for inclusion) and wound dehiscence and drainage (secondary, studies did not have to report on these outcomes for inclusion). Study designs included were randomized controlled trials (RCTs) and prospective or retrospective observational studies (both comparative and noncomparative). The secondary focus was to include studies that reported on SSI and reported/compared dressing use as well as wound closure methods. Studies were excluded based on the following criteria: study designs such as white papers, commentaries, narrative reviews, models; non-English studies including nonhuman, phantom, or cadaver subjects; and studies on fewer than 50 total patients.

A data extraction form was developed in Microsoft Excel, and a single reviewer extracted data for each study; data were checked for accuracy and completeness by a second reviewer with discrepancies resolved by consensus. The following information was extracted: geographic location, study design, closure methods, and dressing types for each study arm by tissue layer (eg, joint capsule/fascia, subcutaneous, subcuticular, superficial skin layer), surgery type, outcome definitions, sample size, deep infections and PJIs, superficial SSIs, prolonged wound drainage, and wound dehiscence.

Key outcomes evaluated in this study include a qualitative summary of the following: methods of wound closure across each tissue layer, superficial and deep SSI rates, and rates of wound drainage and dehiscence. Analyzing findings using a meta-analysis was explored; however, several challenges made such an approach impractical including large heterogeneity in level of details reported for wound closure methods, outcome definitions, and study populations. Thus, this focused literature review systematically presents wound closure methods by layer and the associated rates of superficial and deep SSI, wound dehiscence, and prolonged drainage.

Study quality was assessed according to their design. Two reviewers independently assessed study quality using the Cochrane risk of bias tool for RCTs [13] or the Newcastle-Ottawa Quality Assessment Scale (NOS) for nonrandomized comparative observational studies [14]. For each nonrandomized study, overall quality was determined based on the total scores on the NOS as follows: ≤5, low quality; 6-7, moderate quality; 8-9, high quality [14]. For each RCT, the risk of bias for each domain (sequence generation, allocation concealment, blinding, incomplete outcome data, selective outcome reporting, and other sources of bias) and across all domains was assigned as low, unclear, or high according to the strategy devised by the Cochrane Collaboration as described by Higgins et al. [13]. Differences in study rating between reviewers were resolved through consensus. Studies were not excluded based on methodological quality. Risk of bias for noncomparative studies [[15], [16], [17], [18]] was not evaluated.

## Results

From the literature search, 956 potentially relevant records were identified, of which 888 were excluded during title and abstract screening. Of the 68 full-text articles assessed for inclusion, 40 publications were included in the qualitative systematic review (Fig. 1). A summary of included study characteristics is provided in Table 1. The risk of bias for the 22 included RCTs was assessed using the Cochrane risk of bias tool [13]. All studies (n = 22) had either low or unclear risk of bias for random sequence generation and allocation concealment (Supplement Table 1). Thirteen RCTs had high risk of bias for both lack of blinding participants and personnel or outcome assessors [[19], [20], [21], [22], [23], [24], [25], [26], [27], [28], [29], [30], [31]], and the other 9 had low or unclear risk of bias [[32], [33], [34], [35], [36], [37], [38], [39], [40]]. Two studies had high risk of bias from incomplete outcome data [26,28], and 7 studies had high risk of bias from other sources [[19], [20], [21],^23^,^24^,^28^,^29^]. Overall, 3 studies had low risk, [34,38,40] 6 studies had unclear risk, [32,33,[35], [36], [37],^39^] and 13 studies had high risk of bias [[19], [20], [21], [22], [23], [24], [25], [26], [27], [28], [29], [30], [31]] The risk of bias for the 14 included comparative observational studies was assessed using the NOS scale [14]. Overall, 13 of the 14 studies were of moderate to high quality (6-7 or 8-9 stars, respectively) [[41], [42], [43], [44], [45], [46], [47], [48], [49], [50], [51], [52]], and 2 received 5 stars [53,54] (Supplement Table 2).

**Table 1:** Summary of included studies.

Author	Year	Region	Study design	Surgery type	Overall sample size	Main comparison	Infection definitions
Khan et al. [20]	2006	Australia	RCT	Hip and knee	187	Sutures vs staples vs TSA	All wounds with discharge after the third day were swabbed and cultured. Where cultures were positive or there was clinical evidence of cellulitis, the patients were treated with a course of antibiotics and recorded as having an “infection.” No further definition of superficial and deep. Text stated, “no cases of deep infection”. Infections reported as “early” and “late” were summed.
Singhal and Hussain [16]	2006	UK	Observational	Mixed hip and knee	182	Single-arm staples	Superficial infection, those with positive wound swabs were regarded as wound infections, was treated with antibiotics. Deep infection not defined or mentioned as outcome.
Khurana et al. [15]	2008	UK	Observational	Hip	93	Single-arm TSA	Definition not reported.
Livesey et al. [24]	2009	UK	RCT	Hip	77	TSA vs staples	Self-reported infection which required antibiotics. Further definition for superficial and deep infection not reported.
Eickmann and Quane [45]	2010	USA	Observational	Knee	165	Traditional sutures and TSA vs barbed sutures and TSA	Definition not reported.
Fisher et al. [26]	2010	USA	RCT	Hip	60	Absorbable staples vs metal staples	Definition not reported.
Miller and Swank [47]	2010	USA	Observational	Hip, knee, and mixed	459	TSA vs staples	Superficial infection defined as requiring antibiotics. Deep infection defined as requiring debridement.
Eggers et al. [22]	2011	USA	RCT	Knee	75	Staples vs TSA vs sutures	Infections categorized into categories 1-3. Infections never exceeded category 1 or superficial infection. Total infections extracted due to report of chi-square.
Newman et al. [53]	2011	USA	Observational	Knee	181	Sutures vs staples	CDC criteria: superficial/incisional, defined as involving only skin and subcutaneous tissue of the incision; deep incisional defined as involving peri-incisional deep soft tissues (eg, fascial and muscle layers); and organ/space defined as involving any part of the body, excluding the skin incision, fascia, or muscle layers, that was opened or manipulated during the operative procedure.
Gililland et al. [42]	2012	USA	Observational	Knee	183	Barbed sutures and staples vs traditional sutures and staples	Superficial infection treated with irrigation and debridement, no other definitions reported.
Patel et al. [48]	2012	USA	Observational	Mixed hip and knee	278	Absorbable sutures and TSA vs nonabsorbable sutures and TSA vs staples	Superficial and wound infection not defined; wound infection was recorded as deep infection. Both reported infections treated with irrigation and debridement.
Ting et al. [38]	2012	USA	RCT	Mixed hip and knee	60	Barbed sutures and TSA vs traditional sutures and TSA	Superficial infection was defined by need for reoperation and/or a more than 10-d course of oral or intravenous antibiotics. Deep infection was defined by positive cultures obtained at the time of reoperation.^a^
Gililland et al. [33]	2014	USA	RCT	Knee	394	Barbed sutures vs traditional sutures. Various skin closure methods used	Hollander Wound infection Grade: The infection grade ranged from no infection, to simple stitch abscess, to surrounding cellulitis, to accompanying lymphangitis, to systemic symptoms. Note to Table 4 from the study describes grade 4 systemic symptoms as deep infection. Reported for 2 and 6 weeks. Four superficial (2 grade 1, 2 grade 2) and 1 deep infections at 6 wk.
Smith et al. [39]	2014	USA	mixed	Mixed hip and knee	134	Barbed sutures vs traditional sutures	Superficial infections are defined as superficial cellulitis without infection deep to the fascia.
Buttaro et al. [21]	2015	Argentina	RCT	Hip	220	Sutures vs staples	Patients were monitored for superficial and deep infections, no further definitions reported.
Maheshwari et al. [44]	2015	USA	Observational	Knee	190	Barbed sutures and staples vs traditional sutures	Definition not reported.
Sah [34]	2015	USA	RCT (randomized by knee in bilateral surgeries)	Knee	100	Barbed sutures vs traditional sutures	Wounds monitored for superficial and deep infections, not further defined no antibiotics or surgical interventions after.^b^
Chow [17]	2016	USA	Observational	Knee	92	Single-arm barbed sutures and microcurrent dressings	Periprosthetic joint infection (did not specify superficial or deep).^c^
Wyles et al. [23]	2016	USA	RCT	Knee	45	Absorbable sutures vs nonabsorbable sutures vs staples	Definition not reported.
Austin et al. [41]	2017	USA	Observational	Knee	2482	Barbed sutures vs traditional sutures. Various skin closure methods used	Superficial infections not studied. Deep infection defined according to the MusculoSkeletal Infection Society guidelines.
Chan et al. [32]	2017	Hong Kong	RCT	Knee	109	Barbed sutures and staples vs traditional sutures and staples	CDC criteria.
Glennie et al. [19]	2017	Canada	RCT	Hip	140	Sutures and TSA vs staples	Definition not reported.
Ko et al. [46]	2017	South Korea	Observational	Knee	90	Staples vs zipline	Superficial and deep SSI not defined. Recorded data listed as “surgical site infection.”
Takayama et al. [49]	2017	Japan	Observational	Knee	71	Sutures vs staples	Superficial and deep SSI not defined.
Li et al. [36]	2018	China	RCT (randomized by knee or hip)	Mixed hip and knee	168	Barbed sutures and staples vs traditional sutures and staples	Superficial defined as a minor complication which could be handled in the ward. Deep infections were defined as major complications which would require return to the operating room.
Lin et al. [35]	2018	Taiwan	RCT	Knee	102	Antimicrobial sutures and staples vs traditional sutures and staples	Definition not reported.^d^
Liu et al. [43]	2018	China	Observational	Knee	180	Antimicrobial sutures vs traditional sutures	Definition not reported.
Rui et al. [25]	2018	China	RCT	Hip	165	Sutures vs staples	Superficial SSIs were defined as an infection involving skin and subcutaneous tissue, while infections involving deep soft tissue including muscle and/or fascia were diagnosed as deep SSIs.^e^
Sprowson et al. [37]	2018	UK	Quasi-randomized	Mixed hip and knee	2546	Antimicrobial sutures vs traditional sutures	Based on CDC definition. Superficial SSI: occurs within 30 d of surgery, involves only the skin or subcutaneous tissue of the incision and meets at least one of the specified criteria. Deep SSI: SSI involving the deep tissues (ie, fascial and muscle layers), within 30 d of surgery (or 1 y if an implant is in place), and the infection appears to be related to the surgical procedure and meets at least one of the specified criteria.
Gamba et al. [27]	2019	USA	RCT	Knee	85	Barbed sutures vs traditional sutures	Superficial SSIs were defined based on the CDC definition of for superficial incisional surgical site infection.
Sakdinakiattikoon and Tanavalee [29]	2019	Thailand	RCT	Knee	60	Barbed sutures vs traditional sutures	Definition not reported.
Sundaram et al. [30,67]	2019	USA	RCT	Knee	54	TSA + polyester mesh vs staples	Superficial SSIs were defined according to literature (Healy 2013 and Deirmengian 2014)
Yuenyongviwat et al. [51]	2019	Thailand	Observational (case matched)	Knee	288	Traditional sutures vs adhesive strips	Definition not reported.
Akdogan and Atilla [54]	2020	Turkey	Observational	Knee	274	Aquacel Ag vs conventional gauze	Definition not reported.
Anderson et al. [52]	2020	USA	Observational	Knee	347	TSA + polyester mesh vs silver impregnated dressing	Definition not reported.
Feng et al. [40]	2020	China	RCT	Knee	582	Barbed sutures (full-layer) vs barbed sutures (joint capsule), traditional absorbable sutures (joint capsule)	Superficial infections were defined in accordance with CDC criteria for superficial incisional surgical site infection. Deep infections were defined based on the MSIS criteria and required operative management and/or IV antibiotics in our cohort.
Herndon et al. [50]	2020	USA	Observational	Hip	323	TSA + polyester mesh vs silver impregnated dressing	Definition not reported.
Mallee et al. [28]	2020	The Netherlands	RCT	Hip	535	Staples vs absorbable sutures	SSI was defined as an infection involving only the skin or subcutaneous tissue of the incision occurring within 30 d of the operation; AND at least 1 of the following: (1) purulent drainage, with or without laboratory confirmation, from the superficial incision; (2) organisms isolated from an aseptically obtained culture of fluid or tissue from the superficial incision; (3) at least 1 of the following signs or symptoms of infection: pain or tenderness, localized swelling, redness, or heat and superficial incision deliberately opened by surgeon, unless incision is culture-negative; (4) diagnosis of superficial incisional SSI made by the surgeon or attending physician.
Snyder et al. [18]	2020	USA	Observational	Mixed hip and knee	>2000	Single-arm barbed suture and TSA + polyester mesh	Definition not reported.
Sundaram et al. [31]	2020	USA	RCT	Knee	60	Barbed sutures vs traditional sutures	Superficial wound infections were defied as infections of the superficial surface of the wound with no physical examination findings or clinical progression associated with deep infection.

IV, intravenous.

a Ting et al., 2012 stated that "No patient developed …” “deep periprosthetic joint infection after discharge,” implying that "deep infection" and "PJI" are used interchangeably in this study.

b Sah et al., 2015 cites the Ting et al., 2012 study which mentions periprosthetic infection, but no other mentions of PJI.

c Chow et al., 2016 mentioned PJI without further definition.

d Lin et al., 2018 used the term “deep PJI.”

e The introduction of Rui et al., 2018 mentions "deep periprosthetic joint infection", but does not use that term to describe deep infections in the main text.

In total, 22 RCTs, 3 prospective observational studies, 11 retrospective observational studies, and 4 single-arm noncomparative studies were included in this systematic review. Most of the identified studies included TKA cohorts (62.5%), followed by THA cohorts in 25%, and mixed TKA and THA cohorts in 20%. Across included studies, there was substantial variability in the methods used for wound closure, with 6 primary closure categories and 22 unique combinations of methods reported (Table 2). In general, categories for wound closure included (1) conventional methods (traditional sutures for deep tissue closure and staples, sutures, or TSA for skin closure; including 20 RCTs); (2) conventional methods with antimicrobial sutures (including 3 RCTs); (3) traditional sutures for deep tissue closure, barbed sutures for subcuticular closure, and traditional TSA or TSA (2-octyl cyanoacrylate) with polyester mesh for skin closure (including one RCT); (4) one layer of barbed sutures for deep tissue closure and traditional skin closure (staples, sutures, TSA; including 6 RCTs); (5) multilayered deep tissue closure with barbed sutures and traditional skin closure (staples, sutures, TSA; including 5 RCTs); and (6) multilayer barbed sutures for deep tissue closure and TSA (2-octyl cyanoacrylate) with polyester mesh for skin closure (including 3 observational studies). A detailed breakdown of wound closure methods, by treatment arm, for each included study, is reported in Supplement Table 3, Supplement Table 4, Supplement Table 5, for knee, hip, and mixed procedure types, respectively.

**Table 2:** Summary categories and techniques of wound closure methods.

Category/Technique	Fascia suture type	Subcutaneous suture type	Subcuticular suture type	Skin	Number of arms	Overall sample	Deep SSI^a^	Superficial SSI^a^	Prolonged drainage^a^	Wound dehiscence^a^
Category One										
1	Traditional	Traditional		Staples	23	2058	0.0% to 2.0%	0.0% to 14.8%	0.0% to 22.2%	0.0% to 6.7%
2	Traditional	Traditional	Traditional	Staples	3	195	0.0%	0.0% to 3.9%	51.3%	0.0%
3	Traditional	Traditional	Traditional	Traditional sutures & TSA	9	650	0.0% to 3.9%	0.0% to 12.1%	0.9% to 39.5%	0.0% to 3.4%
4	Traditional	Traditional	Traditional	Traditional sutures	17	3928	0.0% to 2.0%	0.0% to 6.7%	0.0% to 16.1%	0.0% to 4.4%
5	Traditional	Traditional		Staples & TSA	1	29	0.0%	10.3%	0.0%	NR
6	Traditional	Traditional	Traditional	Staples & TSA	1	203	0.5%	2.0%	NR	NR
Cateogory Two										
7	AM Traditional	AM Traditional		Staples	1	51	0.0%	0.0%	NR	NR
8	AM Traditional	AM Traditional			1	137	0.0%	1.5%	NR	NR
9	Traditional (±AM)	Traditional (±AM)	Traditional (±AM)		4	1504	0.0% to 1.1%	0.0% to 1.3%	10.0%	0.0%
Cateogory Three										
10	Traditional	Traditional	Barbed	TSA	1	46	2.2%	0.0%	NR	2.2%
11	Traditional	Traditional	Barbed	TSA + polyester mesh	1	30	0.0%	3.0%	NR	3.0%
Cateogory Four										
12	Barbed	Traditional		Staples & TSA	1	31	0.0%	6.5%	0.0%	NR
13	Barbed	Traditional		Staples	4	1182	0.0% to 0.5%	0.0% to 5.0%	0.1%	0.3% to 11.0%
14	Barbed	Traditional	Traditional	Traditional sutures & TSA	2	37	0.0% to 0.0%	21.0% to 33.0%	0.0% to 0.0%	5.0% to 6.0%
15	Barbed	Traditional	Traditional	Traditional sutures	3	99	0.0% to 0.0%	0.0% to 26.0%	0.0%	0.0% to 5.0%
Cateogory Five										
16	Barbed	Barbed		Staples	3	194	0.0%	0.0% to 7.5%	NR	1.0% to 5.0%
17	Barbed	Barbed	Traditional	Staples	1	115	0.0%	NR	NR	0.0%
18	Barbed	Barbed	Barbed	TSA	3	387	0.0% to 0.7%	0.0% to 1.1%	NR	0.6% to 2.2%
19	Barbed	Barbed	Traditional	Staples & TSA	1	191	0.5%	3.1%	NR	NR
20	Barbed	Barbed	Barbed		3	220	0.0% to 2.0%	0.0% to 6.1%	6.7%	0.0% to 8.2%
21	Barbed	Barbed	Barbed	Barbed sutures	1	193	2.1%	9.8%	NR	NR
Cateogory Six										
22	Barbed	Barbed	Barbed	TSA + polyester mesh	3	>2362	0.0% to 1.1%	0.0%	NR	0.0%

Note: Studies in each technique with “NR” outcomes were not captured in the presented ranges, please see Supplement Table 3, Supplement Table 4, Supplement Table 5 in the Appendix for article specific outcomes.

AM, antimicrobial sutures; NR, not reported.

a Columns with only one value indicate that only one study reported on this outcome.

The most commonly reported method for wound closure in hip and knee arthroplasties consisted of conventional closure methods (#1), with 7063 patients evaluated across studies. In general, this category for wound closure had large ranges in rates reported for deep SSI (0% to 3.9%) and prolonged drainage (0% to 51.3%). For the other wound-healing complications, superficial SSI ranged from 0% to 14.8%, and wound dehiscence ranged from 0% to 6.7%.

For wound closure categories with more recent technologies, rates of wound complications were reported to be particularly low for the category (#6) of barbed sutures for deep tissue layers and TSA with polyester mesh for skin closure. This category evaluated over 2362 patients and reported 0% to 1.1% deep SSI rates and 0% incidence of superficial SSI or wound dehiscence (Drainage was not reported.). The other category that reported very low rates of adverse event rates included antimicrobial sutures for deep and superficial layers (#2). This category consisted of 1692 patients and reported a narrow range in both deep (0% to 1.1%) and superficial (0% to 1.5%) SSIs. Other evaluated adverse events were poorly reported, with only one study reporting prolonged wound drainage (10%) and wound dehiscence (0%).

Of the remaining 3 categories, the use of traditional sutures for deep tissue and barbed sutures with TSA (with or without polyester mesh; #3) for superficial closure was the least studied, with 76 patients. Adverse event rates were generally low, with deep SSI ranging from 0% to 2.2%, superficial SSI ranging from 0% to 3%, and wound dehiscence ranging from 2.2% to 3% (Prolonged wound drainage was not reported.). Closure categories using barbed sutures for deeper tissue closure in single (#4) or multiple layers (#5) were evenly studied in 1349 and 1300 patients, respectively. The use of barbed sutures for a single deep tissue layer reported a low adverse event range for deep SSI (0% to 0.5%) and a wide range for superficial SSI (0% to 33%). The use of barbed sutures for multiple deep layer closure reported a range of 0% to 2.1% for deep SSI and a range of 0% to 9.8% for superficial SSI. For wound dehiscence, a range of 0% to 8.2% was reported.

When comparing rates of adverse events between procedures, there appeared to be no obvious differences between TKA and THA. The ranges of deep SSI rates overlapped across procedure types and ranged from 0% to 2.1% for TKA, 0% to 2.0% for THA, and 0% to 3.9% for mixed TKA/THA studies. Superficial SSI rates were usually higher than deep SSI rates and ranged from 0% to 33.0% for TKA, 0% to 12.1% for THA, and 0% to 10.3% for mixed TKA/THA studies. Prolonged wound drainage and wound dehiscence were less commonly reported than SSI across procedure types. Prolonged wound drainage rates ranged from 0% to 37% for TKA, 0.9% to 51.3% for THA, and 0% to 1.7% for mixed TKA/THA studies. Wound dehiscence rates ranged from 0% to 11.0% for TKA, 0% to 1.9% for THA, and 0.6% to 8.2% for mixed TKA/THA studies. For most studies that reported prolonged wound drainage and wound dehiscence, the rates ranged from 0% to 10%, but there were a few studies with much higher rates of prolonged wound drainage for TKA [20] and THA [24,26].

When reviewing adverse event rates across categories and techniques based only on RCTs, findings generally aligned with the wider study inclusion set (Appendix D); however, data were not available for certain categories (eg, #6, multilayer barbed sutures with TSA and polyester mesh) and techniques (eg, #18 multilayer barbed suture and TSA alone).

## Discussion

In summary, the methods of wound closure across tissue layers varied highly, with 6 primary closure categories and 22 unique technique combinations noted in the literature. These findings highlight the substantial variability that exists in wound closure methods for hip and knee arthroplasties, with varying rates in adverse events. In addition, this review identified considerable heterogeneity across studies for the level of detail used to describe closure, the population evaluated, and definitions used when reporting outcomes. Therefore, comparison between closure techniques or categories has been limited to a qualitative summary as meta-analyses were deemed to be inappropriate. Overall, each of the identified categories for closure methods reported low rates of adverse events, with many reporting zero incidences. In addition, there appeared to be no obvious differences between studies evaluating TKA, THA, or mixed procedures for rates of adverse events. However, some differences were observed between adverse event ranges by categories of wound closure which are highlighted in the following sections.

SSIs were the most common wound complications reported in the studies included in this review. Conventional closure methods, with traditional sutures for deep tissue layers and sutures, staples, or TSA for superficial closure (category #1), had high variability in the rate of deep SSI (0% to 3.9%) and superficial SSI (0% to 14.8%). Wound closure categories which reported very low SSI rates with very narrow ranges included the category of multilayer antimicrobial sutures (category #2), as well as the category of barbed sutures for deep tissue layers with TSA and polyester mesh for skin layer (category #6). Across these 2 categories, over 3000 patients were studied, the risk of deep SSI ranged from 0% to 1.1%, and the risk of superficial SSI ranged from 0% to 1.3%. For superficial SSI, the closure category with high variability in rates was barbed sutures for a single layer with traditional skin closure (0% to 33%) (category #4), although sample size may have contributed to this large range as the studies reporting the higher rates included only 18 to 36 patients in each treatment arm with various infection definitions [19,21,37]. Collectively, these findings are important as SSIs are a serious adverse event that contributes to increased hospital stay, early readmission rates, mortality, and excess hospital costs [55].

Prolonged wound drainage was one of the least reported outcomes across included studies; however, rates were the highest of the outcomes studied (up to 51%). The majority of the data reporting prolonged drainage came from the category of conventional wound closure methods (category #1), with drainage rates ranging from 0% to 51%. Other wound closure categories typically reported prolonged wound drainage rates of 10% or less; however, data were not available for several techniques. Reductions in prolonged drainage is an important factor in wound healing as it has been found to be a key risk factor for infection, with up to 12 times higher risk of infection associated with prolonged drainage lasting greater than 5 days [56]. Similarly, wound dehiscence was poorly reported across identified studies. Across closure categories, wound dehiscence rates generally ranged between 0% and 10%; however, there were limitations in data reporting for several techniques. It is noted that 2 of the 6 categories (ie, antimicrobial sutures [#2] and multilayer barbed sutures with TSA and polyester mesh [#6]) reported rates of 0% from 2 studies. Overall, these findings highlight an important area for future study as wound dehiscence is a serious adverse event that can lead to other complications, including risk of early readmission [57]. Both wound dehiscence and prolonged drainage are important considerations that delay wound healing [50,52]. Delayed wound healing has been noted to be a leading risk factor for PJI/SSI, patient morbidity, and increased health-care costs [50,52].

Results of this review highlight the need for use of consistent, uniform, and watertight multilayer closure methods to avoid adverse events and unnecessary readmissions. Across the included studies, key categories reporting very low adverse event rates included the use of antimicrobial sutures (category #2) and barbed sutures for deep tissue with TSA and polyester mesh for skin closure (category #6). The first category of closure using antimicrobial sutures is aligned with the wealth of evidence highlighting their benefit in reducing the risk of SSI. This includes multiple, large, meta-analyses and multinational guidelines recommending their use across surgery types (ie, World Health Organization, CDC, National Institute for Health and Care Excellence, and so forth) [8,[58], [59], [60], [61]]. For the second category of closure with barbed sutures for deep closure and TSA with polyester mesh for superficial skin layer, the low rate of wound closure–related adverse events observed may be due to various factors. The use of barbed sutures for deep tissue closure may allow watertight closure of the tissue over a shorter operation duration due to their ability to eliminate the need to tie surgical knots and knot-related complications [18,31]. The benefits of TSA with polyester mesh for superficial skin closure to reduce the risk of adverse events may include its strength (equivalent to 3-0 suture), tension-sharing properties (evenly distributing tension across the width of the mesh instead of at individual anchor points), and mechanical barrier properties (may prevent entry of 99% of pathogens over the wound) [50,52].

A recent, large observational study by Snyder et al. reported on the use of watertight multilayer closure with recent technologies, examining the role of barbed sutures and TSA with a polyester mesh as part of an integrated clinical pathway (ICP) [18]. The study was conducted in over 2000 registry-verified primary hip and knee arthroplasties to simultaneously address multiple adverse events [18]. In knee arthroplasty, closure included specific products and techniques for the synovium, joint capsule, subcutaneous, subcuticular, and final layers. In hip arthroplasty, closure included specific products and techniques for the joint capsule, hip bursa, iliotibial band, subcutaneous, subcuticular, and final layers. By implementing systematic and comprehensive ICPs with multilayer, watertight closure, Snyder et al. found improved outcomes compared with historical conventional methods, with zero transfusion, no injurious hospital falls, no SSIs, no serious 90-day opioid complications, no early hip dislocations, and fewer than 0.1% venous thromboembolism-related readmissions [18]. In addition, the program found total per-episode cost of care was reduced by more than 20% due to lower length of stay and readmissions, verified by a formal Centers for Medicare & Medicaid Services comparison [18]. These findings highlight the potential benefits of clinical pathways that focus on consistent, uniform, and watertight multilayer closure.

In addition, optimizing wound closure is an important factor to improve patient outcomes and reduce health-care costs given current health-care reform and bundled payment initiatives established by Centers for Medicare & Medicaid Services for hospital reimbursement of hip and knee arthroplasties [62]. Implementing a patient pathway incorporating multilayer, watertight closure, such as the ICPs that have demonstrated low rates of adverse events or delayed wound-healing complications, can enable hospitals to avoid excess costs given that high rates of adverse events will unfavorably impact bundled payments. Another important consideration is the impact of different closure methods on value-based purchasing programs such as the Hospital Readmissions Reduction Program [63]. Adverse events associated with delayed wound healing, such as SSI, wound dehiscence, and prolonged drainage, may impact the stress experienced by patients and providers, potentially diminishing the benefit of these programs [52].

The findings of this qualitative systematic review are aligned with the findings from existing reviews; however, several important differences exist. In general, both this study and previous literature identified a wide range of wound closure adverse events reported across studies [[10], [11], [12],[64], [65], [66]]. Key strengths of this study, compared with past publications, is the more comprehensive, systematic approach taken for identifying and categorizing closure methods, with detailed techniques used within different tissue layers—from joint capsule/fascia to superficial layer—being summarized to present a more complete assessment of wound closure after hip and knee arthroplasties. Past studies have not comprehensively assessed all different types of wound closure methods and products used in practice in a single review.

As a result of the comprehensive inclusion of various studies, a key limitation of this systematic review was the inability to pool evidence in a meta-analysis. This was due to substantial heterogeneity across studies for methods of closure by tissue layer, product types used within layers, other factors impacting SSIs and PJIs, lack of consistent SSI definitions, and absent standardized reporting. Furthermore, many of the recently introduced technologies (eg, TSA with polyester mesh) and wound closure methods have relatively limited data compared with conventional methods. Finally, the quality assessment in our study indicated that several randomized studies were associated with high risk of bias. Although a large source of this bias appeared to be suboptimal study blinding, due to inherent challenges associated with blinding of devices and supplies, several assessments had additional sources of bias which could not be clearly elucidated. As such, additional high-quality, comparative studies that comprehensively assess promising technologies with early reports of low rates of wound-healing-related adverse events are needed.

## Conclusions

There is a need to standardize methods for wound closure in hip and knee arthroplasties to minimize the risk of complications after the procedure. The optimal tools available to powerfully mitigate practice variations may include ICPs in conjunction with multilayer, watertight closure because they standardize important perioperative best practices impacting multiple adverse events. The use of multilayer watertight closure technologies, including, antimicrobial sutures, barbed sutures, and TSA with polyester mesh, has demonstrated very low rates of wound-healing complications, such as SSIs and delayed wound healing, in patients who underwent primary hip or knee arthroplasty.

## Conflicts of interest

The authors declare the following financial interests/personal relationships which may be considered as potential competing interests: Mark A. Snyder is a paid consultant of Ethicon, Inc. Brian P. Chen is an employee of Ethicon, Inc. George W.J. Wright and Andrew Hogan are employees of CRG-EVERSANA, which received funding from Ethicon, Inc.

## Figures and Tables

**Supplement Table 1:** Methodological quality assessment of the RCT using the Cochrane risk of bias tool.

Study	Random sequence generation	Allocation concealment	Blinding of participants and personnel	Blinding of outcome assessors	Incomplete outcome data	Selective reporting	Other bias	Summary assessment
Chan, 2017 [32]	Low	Unclear	Low	Low	Low	Low	Low	Unclear
Gililland, 2014 [33]	Unclear	Unclear	Low	Unclear	Low	Low	Low	Unclear
Sah, 2015 [34]	Low	Low	Low	Low	Low	Low	Low	Low
Lin, 2018 [35]	Low	Low	Low	Low	Low	Unclear	Low	Unclear
Li, 2018 [36]	Low	Unclear	Low	Low	Low	Low	Low	Unclear
Glennie, 2017 [19]	Low	Unclear	High	High	Low	Low	High	High
Khan, 2006 [20]	Low	Low	High	High	Low	Low	High	High
Buttaro, 2015 [21]	Low	Unclear	High	High	Unclear	Unclear	High	High
Eggers, 2011 [22]	Low	Unclear	High	High	Low	Low	Low	High
Wyles, 2016 [23]	Low	Low	High	High	Low	Low	High	High
Livesey, 2009 [24]	Low	Low	High	High	Low	Low	High	High
Sprowson, 2018 [37]	Low	Unclear	Low	Low	Low	Low	Unclear	Unclear
Rui, 2018 [25]	Unclear	Low	High	High	Low	Low	Low	High
Fisher, 2010 [26]	Unclear	Low	High	High	High	Low	Low	High
Ting, 2012 [38]	Low	Low	Low	Low	Low	Low	Low	Low
Smith, 2014 [39]	Low	Low	Low	Unclear	Low	Low	Low	Unclear
Feng, 2020 [40]	Low	Low	Low	Low	Low	Low	Low	Low
Gamba, 2020 [27]	Low	Low	High	Unclear	Low	Low	Low	High
Mallee, 2020 [28]	Low	Unclear	High	Low	High	Low	High	High
Sakdinakiattikoon, 2019 [29]	Low	Unclear	High	Unclear	Low	Low	High	High
Sundaram, 2019 [30]	Low	Low	High	High	Low	Low	Low	High
Sundaram, 2020 [31]	Low	Low	High	Low	Low	Low	Low	High

**Supplement Table 2:** Methodological quality assessment of observational studies using the Newcastle-Ottawa Quality Assessment Scale.

Study	Representativeness of exposed cohort^a^	Selection of the nonexposed cohort^a^	Ascertainment of exposure^b^	Demonstration that outcome of interest was not present at the start of the study^a^	Comparability of the cohorts on the basis of design or analysis^c^	Assessment of outcome^d^	Was the follow-up long enough for outcomes to occur^e^?	Adequacy of follow-up of cohorts^f^	Total
Austin, 2017 [41]	1	1	1	1	1	1	1	1	8
Eickmann, 2010 [45]	1	1	1	1	1	1	1		7
Gililland, 2012 [42]	1	1	1	1	1	1	1	1	8
Ko, 2017 [46]	1	1		1	1	1	1	1	7
Liu, 2018 [43]	1	1	1	1	1	1	1	1	8
Maheshwari, 2015 [44]	1	1	1	1	1	1	1	1	8
Miller, 2010 [47]	1	1	1	1	1	1	1		7
Newman, 2011 [53]	1	1	1	1		1			5
Patel, 2012 [48]	1	1	1	1		1	1		6
Takayama, 2017 [49]	1	1	1	1	1		1		6
Akdogan, 2020 [54]	1	1	1	1	1				5
Anderson, 2020 [52]	1	1	1	1	1	1	1		7
Yuenyongviwat, 2019 [51]	1	1	1	1	1	1	1		7
Herndon, 2020 [50]	1	1	1	1	1		1		6

a All studies received a star for representativeness of the exposed cohort, selection of the non-exposed cohort, and demonstration that the outcome of interest was not present at the start of the study.

b All but one study received a star for ascertainment of exposure using surgical records and the study that did not included no description of the ascertainment of exposure [10.1016/j.jss.2020.11.087].

c Twelve of the studies received one star for comparability of the cohorts on the basis of design or analysis [[41], [42], [43], [44], [45], [46], [47], [49], [50], [51], [52], [54]] since they did not perform adjusted analyses, regression, or matching for the outcomes of interest. The cohorts from most studies were balanced in terms of patient age and other baseline characteristics apart from those of two studies [48,53].

d All but two of the studies received a star for the assessment of the outcome with record linkage [49,54].

e Only two studies did not follow patients for at least one month following surgery [53,54] and did not receive a star for follow-up duration long enough for outcomes to occur.

f Five studies reported either no loss to follow-up or follow-up of >80% of patients [[41], [42], [43], [44],^46^] and received a star, the other nine studies did not provide information of loss to follow-up [45,[47], [48], [49], [50], [51], [52], [53], [54]].

**Supplement Table 3:** Summary study details for wound closure methods by tissue layer in knee arthroplasty.

Study	Sample size	Fascia		Subcutaneous		Subcuticular		Skin							SSI		Prolonged drainage	Wound dehiscence
		TS	BS	TS	BS	TS	BS	TS	BS	Staples	TSA	TSA/Mesh	Dressing	Other	Deep	Superficial		
Khan et al., 2006 [20]^,^^a^	27	●		●						●			●		0.0%	14.8%	22.2%	0.0%
	27	●		●		α					D		●		0.0%	11.1%	37.0%	0.0%
	31	●		●		●		α					●		0.0%	6.5%	16.1%	0.0%
Eickmann and Quane, 2010 [45]^,^^b^	86	●		●		●		α			D		T		1.1%	1.1%	NR	3.4%
	79		Q		Q						D		T		0.0%	1.1%	NR	2.2%
Miller and Swank, 2010 [47]^,^^c^	93	●		●						●					0.0%	4.3%	0.0%	0.0%
	143	●		●		●		α			D				0.0%	2.8%	2.8%	0.7%
Eggers et al., 2011 [22]^,^^d^	19		Q	●						●					0.0%	5.0%	NR	11.0%
	19		Q	●		α		α			D				0.0%	21.0%	NR	5.0%
	18		Q	●		α		α			H				0.0%	33.0%	NR	6.0%
	19		Q	●		α		●							0.0%	26.0%	NR	5.0%
Newman et al., 2011 [53]^,^^e^	82	●		●						●			●		0.0%	0.0%	0.0%	0.0%
	99	●		●		α		α					●	SS	2.0%	2.0%	1.0%	3.0%
Gililland et al., 2012 [42]	85	●		●						●			●		NR	0.0%	NR	1.2%
	98		Q		Q					●			●		NR	1.0%	NR	1.0%
Gililland et al., 2014 [33]^,^^f^	203	●		●		●		α		●	D			SS	0.5%	2.0%	NR	NR
	191		Q		Q	●		α		●	D			SS	0.5%	3.1%	NR	NR
Maheshwari et al., 2015 [44]	75	●		●		E		α					X		0.0%	NR	NR	1.3%
	115		Q		Q	●				●			X		0.0%	NR	NR	0.0%
Sah, 2015 [34]^,^^g^	50	●		●		●		α					●		0.0%	0.0%	0.0%	0.0%
	50		Q	●		●		α					●		0.0%	0.0%	0.0%	0.0%
Chow, 2016 [17]	92		Q		Q		Q						MD	SS	NR	0.0%	NR	1.1%
Wyles et al., 2016 [23]^,^^h^	15	●		●						●			●		NR	0.0%	NR	NR
	15	●		●		N		N					●		NR	6.7%	NR	NR
	15	●		●		●		α					●		NR	0.0%	NR	NR
Austin et al., 2017 [41]^,^^i^	1598	●		●		α		α		α				SP	0.0%	NR	0.0%	0.0%
	884		S	●		α				α				SP	0.2%	NR	0.1%	0.3%
Chan et al., 2017 [32]	55		S		S					●			●		0.0%	0.0%	NR	1.8%
	54	●		●						●			●		0.0%	1.9%	NR	5.6%
Ko et al., 2017 [46]^,^^j^	45	α		●						●			●		NR	6.7%	NR	6.7%
	45	α		●		α							●	Z	NR	2.2%	NR	4.4%
Takayama et al., 2017 [49]^,^^k^	37	α		●						●			●		0.0%	0.0%	NR	NR
	34	α		●		α		α					●	SS	0.0%	0.0%	NR	NR
Lin et al., 2018 [35]	51	●		●		●				●					0.0%	3.9%	NR	NR
	51	AM		AM		AM				●					0.0%	0.0%	NR	NR
Liu et al., 2018 [43]^,^^l^	80	●		●		●		α							NR	1.3%	NR	1.3%
	100	●		●		AM		AM							NR	0.0%	NR	0.0%
Gamba et al., 2019 [27]	44	●		●						●					0%	2.30%	NR	2.30%
	41		Q		Q					●					0%	7.50%	NR	5%
Sakdinakiattikoon and Tanavalee, 2019 [29]^,^^m^	30	AM		AM		AM								LS	0%	0%	10%	0%
	30		Q		Q		Q							LS	0%	0%	7%	0%
Yuenyongviwat et al., 2019 [51]	151	AM		AM		●		α						SS	0.66%	1.32%	NR	NR
	137	AM		AM										SS	0%	1.46%	NR	NR
Sundaram et al., 2019 [30,67]^,^^n^	30	●		●			S					●			0%	3%	NR	3%
	30	●		●						●					0%	0%	NR	0%
Akdogan and Atilla, 2020 [54]^,^^o^	135	α		α						●			A		NR	2.20%	NR	NR
	139	α		α						●			●		NR	3.70%	NR	NR
Anderson, 2020 [52]			Q		α		●					●			0%	0%	NR	0%
			Q		α		●				SwS		A		0%	0%	NR	0.6%
Feng et al., 2020 [40]	193		S		S		S		S						2.10%	9.80%	NR	NR
	195		S	●						●					0.50%	3.10%	NR	NR
	194	●		●						●					1%	4.10%	NR	NR
Sundaram et al., 2020 [31]	30	●		●		●							A	SS	0%	0%	NR	0%
	30		S	●		●							A	SS	0%	0%	NR	0%

α, assumption; A, Aquacel; AM, antimicrobial sutures; BS, barbed sutures; D, Dermabond; E, Ethilon; H, Histoacryl; L, Liquiband; LS, Leukostrip; N, Nylon; NR, not reported; O, Opsite; Q, Quill; S, STRATAFIX; SP, surgeon preference; SS, Steri-strips; SSI, surgical site infection; SwS, SwiftSet; T, Tegaderm; TS, traditional sutures; TSA, topical skin adhesive; X, Xeroform; Z, Zipline.

a Khan, 2006 for the TSA arm assumed that no sutures were used on the subcuticular layer as 2 layers of Dermabond were used. For the traditional sutures arm 3.0 Monocryl was used for "subarticular" closure, superficial closure not mentioned.

b Eickmann, 2010 for the traditional sutures and TSA arm a running stitch was used for subcuticular and assumed that stitch technique for subcuticular would close the skin as well.

c Miller, 2010 for the traditional sutures and TSA arm assumed that stitch technique for subcuticular would close the skin as well.

d Eggers, 2011 explicit definition for superficial infection not reported in the study, however, "superficial" terminology was used.

e Newman, 2011 for the Steri-strips arm a running subcuticular technique was used and assumed that stitch technique for subcuticular would close the skin as well.

f Gililland, 2014 various superficial subcuticular closure methods used: staples, sutures, TSA, or Steri-strips. Assumed that subdermal sutures would also close the skin if used alone.

g Sah, 2015 a running stitch was used for subcuticular closure in both arms and assumed that stitch technique for subcuticular would close the skin as well.

h Wyles, 2016 for the Nylon suture group a vertical mattress technique with 2-0 nylon suture was used which would close both the subcuticular and skin layers. For the traditional suture group, a running subcuticular technique was used which would close both the subcuticular and skin layers.

i Austin, 2017 surgeon preference was used for skin closure and the devices were not specified. Assumed that the method used for skin closure chosen by the surgeon would close the subcuticular layer as well.

j Ko, 2017 the fascia/joint capsule closure method was not specifically stated for both arms. Assumed that an additional subcuticular closure method would not be used with Zipline closure.

k Takayama, 2017 the fascia/joint capsule closure method was not specifically stated for both arms. Assumed that subcuticular/superficial sutures would not have been used with Steri-strips.

l Liu, 2018 for the traditional sutures arm a vertical mattress technique was used with Mersilk non-absorbable sutures. For the antimicrobial sutures arm a running subcuticular technique was used. For both arms assumed that stitch technique for subcuticular would close the skin as well.

m Sakdinakiattikoon, 2019 postoperative drainage from the incision was reported, assumed this was equal to ‘prolonged drainage’.

n Sundaram, 2019 sample size reported value represents the number of knees treated; total study sample size was 54 patients.

o Akdogan, 2020 surgical site was closed with interrupted sutures; assumed that sutures were used for all layers.

**Supplement Table 4:** Summary study details for wound closure methods by tissue layer in hip arthroplasty.

Study	Sample size	Fascia		Subcutaneous		Subcuticular		Skin							SSI		Prolonged drainage	Wound dehiscence
		TS	BS	TS	BS	TS	BS	TS	BS	Staples	TSA	TSA/Mesh	Dressing	Other	Deep	Superficial		
Khan et al., 2006 [20]^,^^a^	36	●		●						●			●		0.0%	8.3%	13.9%	0.0%
	33	●		●		α					D		●		0.0%	12.1%	12.1%	0.0%
	33	●		●		●		α					●		0.0%	0.0%	9.1%	0.0%
Khurana et al., 2008 [15]^,^^b^	93	●		●		●		α			D				0.0%	0.0%	2.2%	1.1%
Livesey et al., 2009 [24]^,^^c^	39	●		●		●				●			O		NR	2.6%	51.3%	0.0%
	38	●		●		●					L		O		NR	2.6%	39.5%	0.0%
Fisher et al., 2010 [26]^,^^d^	30	●		●						I			●	SS	0.0%	0.0%	3.0%	NR
	30	●		●						●			●	SS	0.0%	0.0%	23.0%	NR
Miller and Swank, 2010 [47]^,^^e^	107	●		●		●		α			D				0.0%	3.7%	0.9%	1.9%
Buttaro et al., 2015 [21]^,^^f^	105	●		●		●				●			●		0.0%	0.0%	NR	4 cases (arm NR)
	115	●		●		●		α					●		0.8%	0.0%	NR	
Glennie et al., 2017 [19]^,^^g^	68	●		●						●			T		1.5%	NR	NR	NR
	72	●		●		●		●			D		T	SS	1.4%	NR	NR	NR
Rui et al., 2018 [25]^,^^h^	83	●		●						●			●		0.0%	2.4%	NR	0.0%
	82	●		●		●		●					●		0.0%	0.0%	NR	0.0%
Herndon et al., 2020 [50]	186		●		α		●					●			1.08%	NR	NR	NR
	137		●		α		●				SwS		A		0.73%	NR	NR	NR
Mallee et al., 2020 [28]	267	α		●		α		●							1%	1%	4%	0.40%
	268	α		●						●					2%	4%	15%	1%

α, assumption; A, Aquacel; BS, barbed sutures; D, Dermabond; I, Insorb; L, Liquiband; NR, not reported; O, Opsite; SS, Steri-strips; SSI, surgical site infection; SwS, SwiftSet; T, Tegaderm; TS, traditional sutures; TSA, topical skin adhesive; V, Vicryl.

a Khan, 2006 rates presented reflect culture positive wounds after patients were discharged as they were treated with a course of antibiotics. However, in the results it was noted one patient with OCA had a superficial wound infection requiring debridement. No cases of deep infection were specifically noted. For the TSA arm, it was assumed that no sutures were used on the subcuticular layer since 2 layers of Dermabond were used. For the traditional sutures arm, 3.0 Monocryl was used for "subarticular" closure and superficial closure was not mentioned.

b Khurana, 2008 assumed that stitch technique for subcuticular would close the skin as well.

c Livesey, 2009 for the TSA arm assumed that the two-step Liquiband adhesive process would not be used with skin sutures.

d Fisher, 2010 used 2-week follow-up value for prolonged drainage rates.

e Miller, 2010 assumed that stitch technique for subcuticular would close the skin as well.

f Buttaro, 2015 the more superficial layer closed with intradermal polypropylene and assumed that intradermal continuous technique would close the skin as well.

g Glennie, 2017 for the TSA arm used uninterrupted subcuticular Monocryl for the dermal/epidermal layer.

h Rui, 2018 for the traditional suture arm running stitch was used for subcuticular closure and assumed that the stitch technique would close the skin as well.

**Supplement Table 5:** Summary study details for wound closure methods by tissue layer in mixed knee and hip arthroplasty.

Study	Sample size	Fascia		Subcutaneous		Subcuticular		Skin							SSI		Prolonged drainage	Wound dehiscence
		TS	BS	TS	BS	TS	BS	TS	BS	Staples	TSA	TSA/Mesh	Dressing	Other	Deep	Superficial		
Singhal and Hussain, 2006 [16]^,^^a^	182	α		α						●			●		NR	0.6%	NR	0.6%
Miller and Swank, 2010 [47]	116	●		●						●					0.0%	0.9%	1.7%	1.7%
Patel et al., 2012 [48]^,^^b^	181	●		●						●					0.6%	0.6%	NR	NR
	51	●		●		●		α			M		X	SS	3.9%	0.0%	NR	2.0%
	46	●		●			VL				M		X	SS	2.2%	0.0%	NR	2.2%
Ting et al., 2012 [38]^,^^c^	29	●		●		●				●	D				0.0%	10.3%	0.0%	NR
	31		Q		Q		Q			●	D				0.0%	6.5%	0.0%	NR
Smith et al., 2014 [39]^,^^d^	36	●		●		●		α					●		0.0%	2.8%	NR	NR
	98		Q		Q		Q						●		2.0%	6.1%	NR	8.2%
Li et al., 2018 [36]	84	●		●						●			●		0.0%	0.0%	NR	NR
	84		Q	●						●			●		0.0%	0.0%	NR	NR
Sprowson et al., 2018 [37]^,^^e^	1323	●		●		α		α					A		1.6%	0.8%	NR	NR
	1223	AM		AM		α		α					A		1.1%	0.7%	NR	NR
Snyder et al., 2020 [18]	>2000	●	S		S		S					●			0%		NR	NR

α, assumption; A, Aquacel; AM, antimicrobial sutures; BS, barbed sutures; D, Dermabond; E, Ethilon; H, Histoacryl; I, Insorb; L, Liquiband; M, Mastisol; NR, not reported; Q, Quill; S, STRATAFIX; SS, Steri-strips; SSI, surgical site infection; TS, traditional sutures; TSA, topical skin adhesive; VL, V-Loc; X, Xeroform.

a Singhal, 2006 mentioned that wounds were closed in layers but did not specify methods.

b Patel, 2012 used 3-0 monofilament Biosyn for closure. Assumed that stitch technique for subcuticular would close the skin as well.

c Ting, 2012 explicitly defined superficial infection as need for reoperation and/or >10-day course of oral or IV antibiotics. Rates for superficial extracted taken from development of peri-incisional erythema (2-5 weeks postop) and were resolved after 7-10 days antibiotics.

d Smith, 2014 running stitch was used for subcuticular closure in the traditional arm. Assumed that stitch technique for subcuticular would close the skin as well.

e Sprowson, 2018 note that Vicryl was used on either the deep fascia or subcutaneous layer not both, subcuticular and skin closure methods not reported.

**Supplement Table 6:** Summary categories and techniques of wound closure methods for RCTs only.

Category/Technique	Fascia suture type	Subcutaneous suture type	Subcuticular suture type	Skin	Number of arms	Overall sample	Deep SSI^a^	Superficial SSI^a^	Prolonged drainage^a^	Wound dehiscence^a^
One										
1	Traditional	Traditional		Staples	13	963	0.0% to 2.0%	0.0% to 14.8%	13.9% to 22.2%	0.0% to 5.6%
2	Traditional	Traditional	Traditional	Staples	3	195	0.0% to 0.0%	0.0% to 3.9%	51.3%	0.0%
3	Traditional	Traditional	Traditional	Traditional sutures & TSA	4	170	0.0% to 3.9%	2.6% to 12.1%	12.1% to 39.5%	0.0%
4	Traditional	Traditional	Traditional	Traditional sutures	9	638	0.0% to 2.0%	0.0% to 6.7%	0.0% to 16.1%	0.0% to 0.4%
5	Traditional	Traditional		Staples & TSA	1	29	0.0%	10.3%	0.0%	NR
6	Traditional	Traditional	Traditional	Staples & TSA	1	203	0.5%	2.0%	NR	NR
Two										
7	AM Traditional	AM Traditional		Staples	1	51	0.0%	0.00%	NR	NR
8	AM Traditional	AM Traditional			0	0	NA	NA	NA	NA
9	Traditional (±AM)	Traditional (±AM)	Traditional (±AM)		1	30	0.0%	0%	10%	0%
Three										
10	Traditional	Traditional	Barbed	TSA	0	0	NA	NA	NA	NA
11	Traditional	Traditional	Barbed	TSA + polyester mesh	1	30	0.0%	3%	NR	3%
Four										
12	Barbed	Traditional		Staples & TSA	1	31	0.0%	3%	0.0%	NR
13	Barbed	Traditional		Staples	3	298	0.0% to 0.5%	0.0% to 5.0%	NR	11.0%
14	Barbed	Traditional	Traditional	Traditional sutures & TSA	2	37	0.0% to 0.0%	21.0% to 33.0%	NR	5.0% to 6.0%
15	Barbed	Traditional	Traditional	Traditional sutures	3	99	0.0% to 0.0%	0.0% to 26.0%	0.0%	0.0% to 5.0%
Five										
16	Barbed	Barbed		Staples	2	96	0.0% to 0.0%	0.0% to 7.5%	0.0% to 0.0%	1.8% to 5.0%
17	Barbed	Barbed	Traditional	Staples	0	0	NA	NA	NA	NA
18	Barbed	Barbed	Barbed	TSA	0	0	NA	NA	NA	NA
19	Barbed	Barbed	Traditional	Staples & TSA	1	191	0.5%	3.1%	NR	NR
20	Barbed	Barbed	Barbed		1	30	0.0%	0.0%	6.7%	0%
21	Barbed	Barbed	Barbed	Barbed Sutures	1	193	2.1%	9.8%	NR	NR
Six										
22	Barbed	Barbed	Barbed	TSA + polyester mesh	0	0	NA	NA	NA	NA

Note: Studies in each technique with “NR” outcomes were not captured in the presented ranges, please see Supplement Table 3, Supplement Table 4, Supplement Table 5 in the Appendix for article specific outcomes.

AM, antimicrobial sutures; NA, not available; NR, not reported.

a Columns with only one value indicate that only one study reported on this outcome.

## Appendix

### Appendix A

Search Strategy Details

MEDLINE search terms

1) (knee[MeSH Terms] OR knee[All Fields]) AND (replace OR replacement)
2) Total knee arthroplasty OR arthroplasty of the knee[Title/Abstract] OR knee arthroplast∗[Title/Abstract] OR knee replac∗[Title/Abstract] OR knee arthroplasty[Title/Abstract] OR "Arthroplasty, Replacement, Knee"[Mesh]
3) ("hip"[MeSH Terms] OR "hip"[All Fields]) AND (replace OR replacement)
4) Total hip arthroplasty OR "Arthroplasty, Replacement, Hip"[Mesh] OR hip replac∗[tiab] OR hip arthroplast∗[tiab] OR arthroplasty of the hip[tiab]
5) (("octyl 2-cyanoacrylate"[Supplementary Concept]) OR "octyl 2-cyanoacrylate"[All Fields] OR Cyanoacrylate OR Octylcyanoacrylate OR "N-butyl-2-cyanoacrylate" OR "2-octyl cyanoacrylate") AND ("Polyester mesh" OR “adhesive mesh”)
6) Dermabond OR Liquiband OR Exofin OR Prineo OR Aquacel OR (Aquacel AND Silver) OR (Aquacel AND Ag) OR Tegaderm OR Opsite OR Sorbact OR "Steri-strip" OR "Steri strip" OR "Skin adhesive" OR "Tissue adhesive" OR suture OR (suture AND barbed) OR (suture AND knotless) staples OR (wound AND closure) OR (wound AND dressing) OR (skin AND closure)
7) 1 OR 2 (search for studies on total knee arthroplasty)
8) 3 OR 4 (search for studies on total hip arthroplasty)
9) 7 OR 8 (total knee arthroplasty OR total hip arthroplasty)
10) 5 OR 6 (closure terms)
11) 9 AND 10 ((total knee arthroplasty OR total hip arthroplasty) AND closure terms)
12) 11 NOT (news[Publication Type] OR comment[Publication Type] OR case reports[Publication Type] OR editorial[Publication Type])
13) 12 with Filters: Humans, English, publication date from January 01, 2000

## Appendix A Supplementary data

Conflict of Interest Statement for Wright

Conflict of Interest Statement for Snyder

Conflict of Interest Statement for Hogan

Conflict of Interest Statement for Chen
