# Supplementary material for: Multilayer Watertight Closure to Address Adverse Events From Primary Total Knee and Hip Arthroplasty: A Systematic Review of Wound Closure Methods by Tissue Layer
Source: Arthroplast Today. 2021 Jul 8;10:180–189.e7. doi: 10.1016/j.artd.2021.05.015 (PMC8430424; doi:10.1016/j.artd.2021.05.015)
Supplement: Conflict of Interest Statement for Snyder [file mmc2.pdf]

CONFLICT OF INTEREST STATEMENT

American Association of Hip and Knee Surgeons

(Adopted from the American Academy of Orthopaedic Surgeons disclosure statement)

The following form **must be filled out completely and submitted by each author (example, 6 authors, 6 forms). All items require a response. If there is no relevant disclosure for a given item, enter "*None.*"**

Multi-layer watertight closure to address adverse events for primary total knee and hip arthroplasty: A systematic review of wound closure methods by tissue layer

Manuscript Title

1. Royalties from a company or supplier (The following conflicts were disclosed)

None

2. Speakers bureau/paid presentations for a company or supplier (The following conflicts were disclosed)

None

3A. Paid employee for a company or supplier (The following conflicts were disclosed)

None

3B. Paid consultant for a company or supplier (The following conflicts were disclosed)

Consultant for Ethicon Inc.

3C. Unpaid consultants for a company or supplier (The following conflicts were disclosed)

None

4. Stock or stock options in a company or supplier (The following conflicts were disclosed)

None

5. Research support from a company or supplier as a Principal Investigator (The following conflicts were disclosed)

None

6. Other financial or material support from a company or supplier (The following conflicts were disclosed)

None

7. Royalties, financial or material support from publishers (The following conflicts were disclosed)

None

8. Medical/Orthopaedic publications editorial/governing board (The following conflicts were disclosed)

None

9. Board member/committee appointments for a society (The following conflicts were disclosed)

None

Each author must sign AND print or type his/her name, date and submit a separate form

In addition, one BLINDED Conflict of Interest form (no author names used) should be submitted per manuscript with all author disclosures.

Mark A Snyder		November 12, 2020
Author Name (Print or Type)	Author Signature	Date
