# Supplementary figures and images for: Multilayer Watertight Closure to Address Adverse Events From Primary Total Knee and Hip Arthroplasty: A Systematic Review of Wound Closure Methods by Tissue Layer
Source: Arthroplast Today. 2021 Jul 8;10:180–189.e7. doi: 10.1016/j.artd.2021.05.015 (PMC8430424; doi:10.1016/j.artd.2021.05.015)

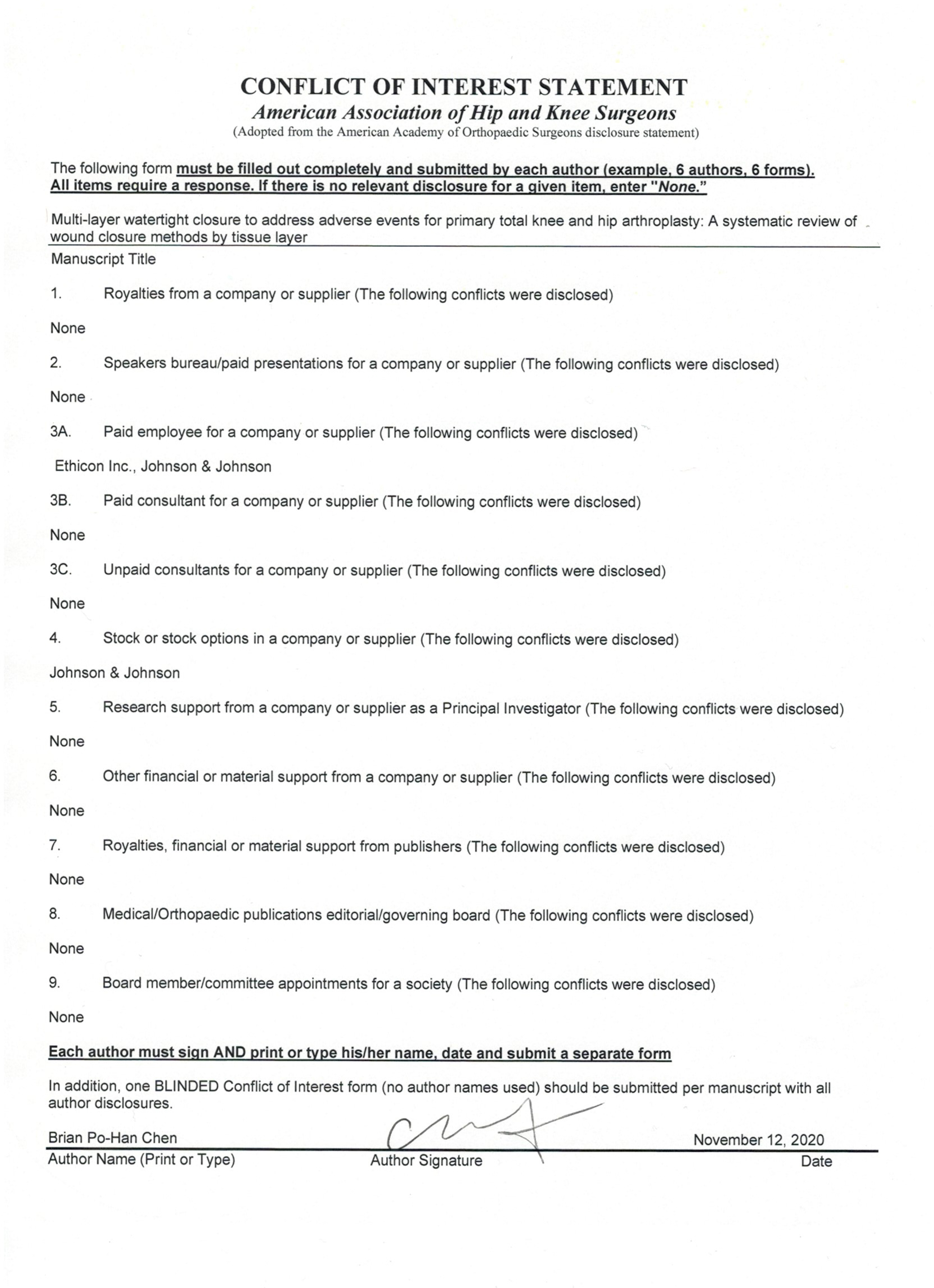

Supplement: Conflict of Interest Statement for Chen [file figs1.jpg]
